# Dynamic PET Imaging Using Dual Texture Features

**DOI:** 10.3389/fncom.2021.819840

**Published:** 2022-01-07

**Authors:** Zhanglei Ouyang, Shujun Zhao, Zhaoping Cheng, Yanhua Duan, Zixiang Chen, Na Zhang, Dong Liang, Zhanli Hu

**Affiliations:** ^1^School of Physics, Zhengzhou University, Zhengzhou, China; ^2^Lauterbur Research Center for Biomedical Imaging, Shenzhen Institute of Advanced Technology, Chinese Academy of Sciences, Shenzhen, China; ^3^Department of PET/CT, The First Affiliated Hospital of Shandong First Medical University, Shandong Provincial Qianfoshan Hospital, Jinan, China

**Keywords:** dynamic PET, texture feature, gray level-gradient cooccurrence matrix (GGCM), gray-level run length matrix (GLRLM), tumor

## Abstract

**Purpose:** This study aims to explore the impact of adding texture features in dynamic positron emission tomography (PET) reconstruction of imaging results.

**Methods:** We have improved a reconstruction method that combines radiological dual texture features. In this method, multiple short time frames are added to obtain composite frames, and the image reconstructed by composite frames is used as the prior image. We extract texture features from prior images by using the gray level-gradient cooccurrence matrix (GGCM) and gray-level run length matrix (GLRLM). The prior information contains the intensity of the prior image, the inverse difference moment of the GGCM and the long-run low gray-level emphasis of the GLRLM.

**Results:** The computer simulation results show that, compared with the traditional maximum likelihood, the proposed method obtains a higher signal-to-noise ratio (SNR) in the image obtained by dynamic PET reconstruction. Compared with similar methods, the proposed algorithm has a better normalized mean squared error (NMSE) and contrast recovery coefficient (CRC) at the tumor in the reconstructed image. Simulation studies on clinical patient images show that this method is also more accurate for reconstructing high-uptake lesions.

**Conclusion:** By adding texture features to dynamic PET reconstruction, the reconstructed images are more accurate at the tumor.

## Introduction

Positron emission tomography (PET) imaging works by imaging an injected radioactive tracer that combines with negative electrons to produce annihilating photons (Zhang et al., 2019; Hu et al., 2020; Zeng et al., 2020). PET imaging provides functional information on a wide range of biochemical and physiological processes (Delcroix et al., 2021; Doyen et al., 2021). To monitor rapid changes in tracer distribution, the scan time per frame is short, thus resulting in poor image quality when images are reconstructed by traditional methods under low-count conditions (Wang and Qi, 2015; Wang, 2019). To improve image quality, scholars have proposed introducing a prior image into PET reconstruction (Green, 1990; Nuyts et al., 2002; Wang and Qi, 2015). However, in the past, when using the information of the prior image, only the intensity information was used, and the texture information of the prior image was ignored. Recently, Gao proposed applying the texture information of the prior image to PET reconstruction (Gao et al., 2021).

Texture features are an important feature of the spatial structure relationship of pixels in an image area (Jiguang, 1984). The term “texture features” is used to describe the surface features of a given object or area (Tang, 1998). Texture features have been widely used in medical image analysis. Initially, texture feature analysis focused mainly on computed tomography (CT) and magnetic resonance imaging (MRI) images that reflect anatomical structure information with higher resolution (Lubner et al., 2017); the main purpose of texture feature analysis was to distinguish tumors from normal tissues. In recent years, there has been an increasing number of studies on texture feature analysis of PET functional metabolism images. Texture information in the PET field has been successfully applied in tumor diagnosis, efficacy evaluation, prognosis prediction, tumor monitoring, genotyping, and pathological typing (Li et al., 2019; Kang et al., 2020; Palumbo et al., 2020; Wady et al., 2020; Zhang J. et al., 2020; Jiang et al., 2021). Machine learning is used to find the distribution of texture features in different groups to achieve accurate judgment and prediction. Additionally, texture features are also used in PET image reconstruction. Gao proposed applying texture features to PET reconstruction and using MRI images as prior images to extract texture features to assist PET reconstruction (Gao et al., 2021).

In this paper, we use two commonly used matrices, namely, the gray level-gradient cooccurrence matrix (GGCM; Jiguang, 1984) and the gray-level run length matrix (GLRLM; Galloway, 1975), to extract texture features and then introduce texture features into dynamic PET reconstruction. The GGCM reflects the two most basic elements of the image, grayscale and gradient. The grayscale reflects mainly the intensity of the image color, and the gradient value is the element that constitutes the outline of the image. The GLRLM reflects the direction, adjacent interval and change of the image grayscale. These two texture features are widely used in computer-aided diagnosis and medical image segmentation (Ren et al., 2020; Soydal et al., 2021). To the best of our knowledge, there are still few studies on the texture feature extraction of radiomics for PET reconstruction, and our incorporation of the GGCM and GLRLM into dynamic PET reconstruction is a new attempt.

The remainder of this article is organized as follows. In section “Materials and Methods”, we introduce the GGCM, GLRLM and proposed method. section “Experiments and Results” describes the computer simulation research and reports the results of simulation experiments. section “Application to Clinical Data” presents the results of applying the new method to real clinical data. We discuss the results in section “Discussion”. Finally, we conclude this article in section “Conclusion”.

## Materials and Methods

### Gray Level-Gradient Cooccurrence Matrix Texture Feature Extraction

An element *H*(*i*, *j*) in the GGCM is defined as the total number of the normalized gray image *F*(*m*, *n*) and the normalized gradient matrix image *G*(*m*, *n*) that have both gray level *i* and gradient *j*(11). The steps to calculate the image gray gradient matrix are as follows.

(1) Obtain the normalized gray matrix

The grayscale image normalization transformation is as follows:


(1) $$F\,\left(K,L\right)=I\,N\,T\,\left(f\,\left(K,L\right)\times N_{H}/f_{M}\right)+1$$

where *N*_H_ is the maximum gray level of the normalized image. In the experiment, *N*_H_ = 8, *f*_*M*_ is the maximum gray level of the original image, and INT represents the rounding operation.

(2) Obtain the normalized gradient matrix

We use the Sobel operator to calculate the gradient value of the pixel:


(2) $$\begin{array}{c} g_{x}=f\,\left(K+1,L-1\right)+2\,f\,\left(K+1,L\right)+f\,\left(K+1,L+1\right)\\ -f\,\left(K-1,L-1\right)-2\,f\,\left(K-1,L\right)-f\,\left(K-1,L+1\right), \end{array}$$

(3) $$\begin{array}{c} g_{y}=f\,\left(K-1,L+1\right)+2\,f\,\left(K,L+1\right)+f\,\left(K+1,L+1\right)\\ -f\,\left(K-1,L+1\right)-2\,f\,\left(K,L-1\right)-f\,\left(K+1,L-1\right), \end{array}$$

(4) $$g\,\left(K,L\right)=\left(g_{x}^{2}+g_{y}^{2}\right)$$

*g* (*K*, *L*) is the gradient value of the (*K*, *L*)th pixel, and then we normalize the gradient image obtained.


(5) $$G\,\left(K,L\right)=I\,N\,T\,\left(g\,\left(K,L\right)\times N_{g}/g_{M}\right)+1$$

where INT represents the rounding operation, *g*_*M*_ represents the maximum gradient value of the gradient image, and *N*_*g*_ represents the maximum gradient value of normalization. In the experiment, *N*_*g*_   = 8.

(3) Statistical GGCM

After obtaining the normalized gray image and the normalized gradient image, the GGCM H is obtained by using mathematical statistics. The meaning of a pixel *H*(*i*, *j*) in the GGCM is the total number of *F* (*K*, *L*) = *i* and *G* (*K*, *L*)   = *j*.

(4) Calculate texture parameters

In the experiment, we used the inverse difference moment of the GGCM, and the calculation formula of the GGCM is as follows:


(6) $$T=\sum_{i=1}^{N_{g}}\sum_{j}^{N_{g}}\frac{1}{1+{\left(i-j\right)}^{2}}\,P\,\left(i,j\right)$$

where $P\,\left(i,j\right)=\frac{H\,\left(i,j\right)}{\sum_{i}\sum_{j}H\,\left(i,j\right)}$.

### Gray-Level Run Length Matrix Texture Feature Extraction

The GLRLM reflects the comprehensive image information on direction, adjacent interval and amplitude of change (Galloway, 1975). The texture features of the GLRLM are calculated as follows:

(1) Quantify the image gray level

The grayscale of the original image is quantized to the range of [1, *N*_H_], and the GLRLM obtained after quantization is [*N*_H_,*N*_L_], where *N*_L_ represents the maximum continuous length of a pixel value.

(2) Calculate the GLRLM

Set the direction θ and step size d. In the experiment, set the direction to 0°, 45°, 90°, and 135°, consecutively; and set the step size to 1. Then, the GLRLM matrix is obtained by calculating the total maximum continuous length of each pixel in each direction. The ordinate of the GLRLM represents the pixel value, and the abscissa represents the maximum continuous length. For example, pixel (*i*, *j*) is the total number of pixels with a gray value of *i* and a continuous length of *j* in a certain direction.

(3) Calculate texture feature

In the experiment, we use the GLRLM’s long-run low gray-level emphasis, whose calculation formula is as follows (Dasarathy and Holder, 1991):


(7) $$L\,R\,L\,G\,E=\frac{1}{n_{r}}\,\sum_{i=1}^{N_{H}}\sum_{j=1}^{N_{L}}\frac{p\,\left(i,j\right)\cdot j^{2}}{i^{2}}$$

where *p* is the GLRLM and $n_{\text{r}}=\sum_{i=1}^{N_{H}}\sum_{j=1}^{N_{\text{L}}}p\,\left(i,j\right)$. The mean value of the long-run low gray-level emphasis in the four directions is taken as the final texture feature.

### Proposed Algorithm

In this paper, we combine the intensity of the prior image, the inverse difference moment of the GGCM and the long-run low gray-level emphasis of the GLRLM to form a kernel function.


(8) $$\kappa \,\left(f_{j}^{\prime },f_{l}^{\prime }\right)=\exp\left(-\frac{{||f_{j}^{\prime }-f_{l}^{\prime }||}^{2}}{2\,\sigma^{2}}\right).$$

where $f_{\text{j}}^{\prime }$ and $f_{\text{l}}^{\prime }$ are feature vectors containing the intensity of the prior image, the inverse difference moment of the GGCM and the long-run low gray-level emphasis of the GLRLM of pixels *j* and *l*, respectively.

The kernelized expectation-maximization (KEM) algorithm for dynamic PET reconstruction can be expressed as (Nuyts et al., 2002):


(9) $$\alpha^{n+1}=\frac{\alpha^{n}}{K^{T}\,P^{T}\,1_{M}}\,\left(K^{T}\,P^{T}\,\frac{y}{P\,K\,\alpha^{n}+r}\right)$$

1_M_ is a unit vector of length *M*.

The final image estimate $\hat{x}$ can be derived by $\hat{\alpha }$ :


(10) $$\hat{x}=K\,\hat{\alpha }$$

**FIGURE 1:**
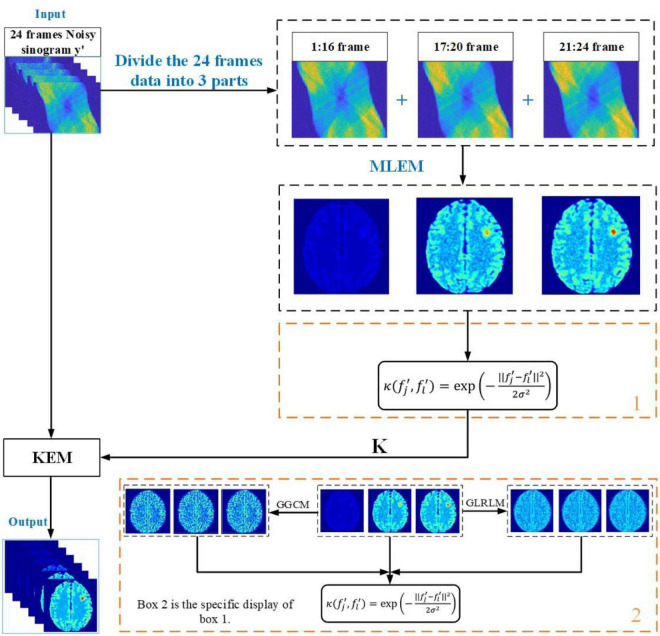
Flowchart of the proposed of algorithm.

The flow chart of the method is shown in Figure 1:

First, we need to obtain prior information, and we divide 24 sinograms into three high-count composite frames according to time; these three frames correspond consecutively to the first 20 min, the middle 20 min and the last 20 min. Then, the maximum likelihood expectation maximization (MLEM) algorithm is used to iterate 100 times on the three composite frames to obtain three prior images. The inverse difference moment of the GGCM and long-run low gray-level emphasis of the GLRLM of each pixel in the three prior images are extracted, and corresponding feature images are generated. Then, the Euclidean distance between the pixel feature vector and the adjacent pixel feature vector is calculated, the kernel function is obtained and applied to the KEM method, and the final image is obtained iteratively. The box labeled 2 in Figure 1 is a specific description of the box labeled 1.

## Experiments and Results

To evaluate the performance of the proposed method, we conducted a computerized simulation experiment and compared the visual effects and quantitative indexes of the images reconstructed by the proposed method with those of the images reconstructed by other methods. The experimental results indicate the feasibility and practicability of the proposed method.

### Digital Phantom Simulations

**FIGURE 2:**
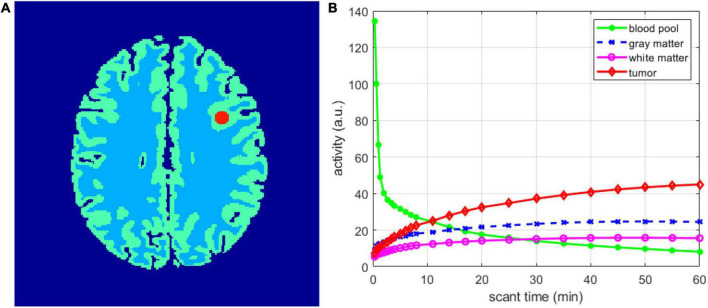
Digital phantom and time activity curves used in the simulation studies. **(A)** 2D brain phantom. **(B)** Regional time activity curves.

In the simulation experiment, we constructed a 2D simulated PET model based on the anatomical model in the Brain Web (Cocosco et al., 1997) database, as shown in Figure 2A. An appropriate axial image of size 217 × 217 pixels was chosen, and the radius of the tumor was 5 pixels. The scanning schedule included the following 24 time frames: 4 × 20 s, 4 × 40 s, 4 × 60 s, 4 × 180 s, and 8 × 300 s. Figure 2B shows the temporal activity curve of the regions. Radiopharmaceuticals were distributed to different brain regions over time. We used a forward projection on a moving image to obtain a noiseless sinogram of 249 × 210 and then introduced Poisson noise. The estimated total number over 60 min is 30 million, including 20% random and scattered events. Attenuation correction was performed in all the reconstruction methods. A total of 10 noisy realizations were simulated, and each realization was independently reconstructed for statistical comparison.

In the computer simulation experiment, in addition to the proposed algorithm, the MLEM method, the KEM method and Gao’s method are also simulated. Gao’s method is called the KEM+ gray-level cooccurrence matrix (GLCM) method in this article. The prior information of the KEM+GLCM method includes the correlation between image intensity and GLCM texture features from prior images. In the simulation experiment, the prior images were reconstructed by sinograms. In terms of considering both the time cost of the computing kernel function and the quality of the reconstructed image, we select the number of neighbors of the searching kernel function as 50. The quantization gray level of the three methods we choose to extract texture features is 8; that is, the pixel value of an image is quantized within the range of 1 to 8. In addition, the neighborhood image size of the GLCM and GGCM is 5 × 5, and the neighborhood image size of the GLRLM is 3 × 3. The neighborhood image size refers to the neighborhood image size centered on a certain pixel, in which the neighborhood image texture features are calculated.

### Performance Metric Evaluation

To measure the quantitative performance of the proposed method, we calculated the signal-to-noise ratio (SNR), normalized mean square error (NMSE), structural similarity (SSIM), standard deviation (SD), and contrast recovery coefficient (CRC).

The SNR is defined as follows:


(11) $$S\,N\,R=10\,\log_{10}\left(\frac{\left|T^{2}\right|}{{\left|\text{T-R}\right|}^{2}}\right)$$

The NMSE is an indicator that reflects the difference between the reconstructed image and the true image and is expressed by


(12) $$N\,M\,S\,E=\frac{\sum_{i=1}^{N}{\left(T_{i}-R_{i}\right)}^{2}}{\sum_{i=1}^{N}{\left(T_{i}\right)}^{2}}$$

where *T* and *R* represent the reconstructed image and true image, respectively; and where N represents the total number of pixels in the reconstructed image.

The SSIM is an index that measures the similarity between the reconstructed image and the true image. When two images are identical, the SSIM equals 1. The SSIM is calculated as follows (Wang et al., 2004):


(13) $$S\,S\,I\,M=\frac{\left(2\,\bar{T}\,\bar{R}+c_{1}\right)\,\left(2\,\sigma_{1}+c_{2}\right)}{\left({\bar{T}}^{2}+{\bar{R}}^{2}+c_{1}\right)\,\left(\sigma_{T}^{2}+\sigma_{R}^{2}+c_{2}\right)}$$

where $\bar{T}$ denotes the average value of the reconstructed image, $\sigma_{R}^{2}$ denotes the variance of the true image, $\sigma_{T}^{2}$ denotes the variance of the reconstructed image, and σ_*1*_ denotes the covariance of the reconstructed image and the true image. *c*_1_ = (*k*_1_L^2^) and *c*_2_ = (*k*_1_L)^2^ are constants used to maintain stability, *k*_1_ = 0.01and *k*_1_ = 0.03,respectively; and L denotes the dynamic range of voxel strength.

To more persuasively demonstrate the effects of the proposed method, we also compared the CRC and SD of images reconstructed by different methods. The SD is calculated by the formula below:


(14) $$S\,D=\frac{1}{\bar{R}}\,\sqrt{\frac{1}{n-1}\,\sum_{i=1}^{N}{\left(T_{i}-R_{i}\right)}^{2}}.$$

The formula for calculating the CRC is as follows:


(15) $$C\,R\,C=\left(\frac{\bar{R}}{{\bar{R}}_{B\,G\,D}}-1\right)/\left(\frac{\bar{T}}{{\bar{T}}_{B\,G\,D}}-1\right)$$

where ${\bar{\text{R}}}_{\text{BGD}}$ denotes the average intensity of the reconstructed image background and ${\bar{T}}_{B\,G\,D}$ denotes the average intensity of the true image background.$\,{\bar{\text{R}}}_{\text{ROI}}$ denotes the average intensity of the region of interest (ROI) of the reconstructed image, and $\,{\bar{\text{T}}}_{\text{ROI}}$ denotes the average intensity of the ROI of the true image.

### Simulation Results

To evaluate the performance of the proposed method, we compared the proposed method with the MLEM method, the KEM method and the KEM+GLCM method. All reconstructions ran for 100 iterations.

**FIGURE 3:**
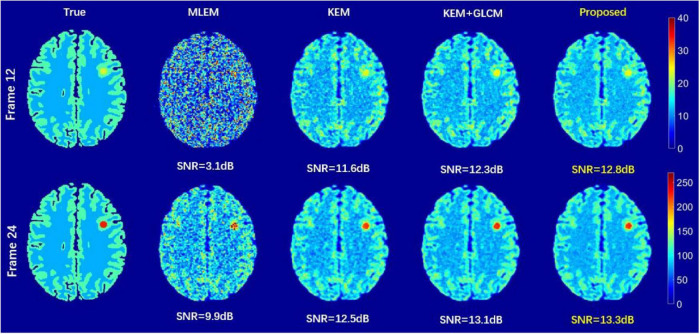
2D brain phantom images reconstructed by different methods.

Figure 3 shows the true images of the 12 and 24th frames and the images reconstructed by the four methods. Frame 12 and frame 24 have 406 k and 2752 k events, respectively. Obviously, the 12th and 24th frames reconstructed by the MLEM algorithm are very noisy. Compared with the SNR of the image reconstructed by the MLEM method, the SNR of the image reconstructed by the KEM method has been improved to a large extent, but the edge preservation is inadequate. Both the KEM+GLCM method and the proposed method are better in terms of preserving edges. Compared with the KEM+GLCM method, the proposed method has higher image quality.

**FIGURE 4:**
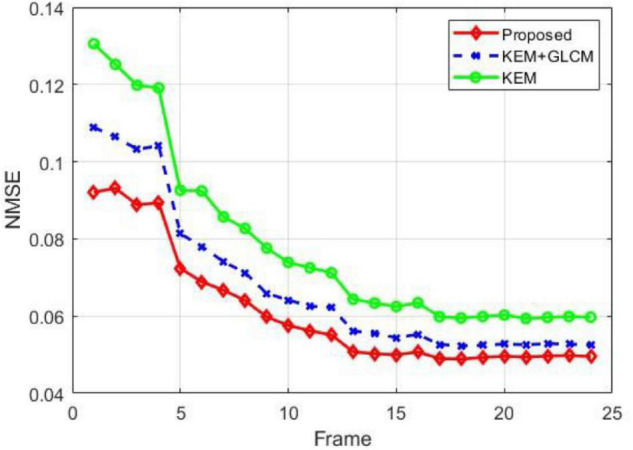
Comparison of the NMSEs of all the frames obtained by different reconstruction methods.

Figure 4 shows the NMSE of the reconstructed images in each time period. Each point represents a time frame. Compared with other methods, the proposed method reduces the noise of the reconstructed images in most time frames. Because the image reconstructed by the MLEM algorithm is too noisy, no comparison is added.

**FIGURE 5:**
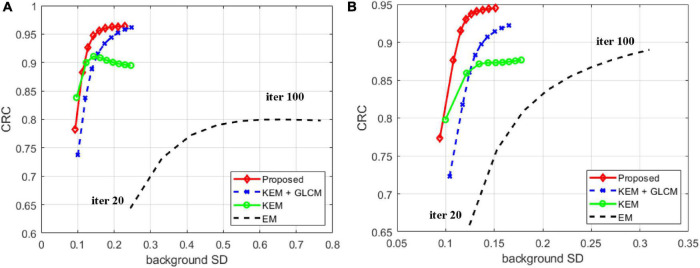
Contrast recovery coefficient of ROIs versus background noise. **(A)** Frame 12. **(B)** Frame 24.

Figure 5 compares the CRC and SD in the white matter region of the tumor with different reconstruction methods in frames 12 and 24. Each point represents ten iterations, and the results show that the proposed method has a high CRC in the tumor area and a low SD in the white matter area.

**FIGURE 6:**
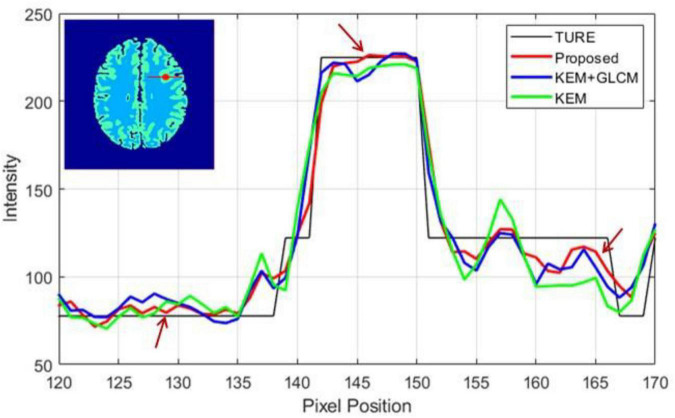
Intensity distribution along a straight line (red) through the tumor area.

Figure 6 shows the relationship between pixel position and pixel intensity for the different methods. A comparison reveals that the image reconstructed by the proposed method is most similar to the contours of the true images and has the highest matching degree. The images reconstructed by the three algorithms all underwent 100 iterations.

## Application to Clinical Data

### Clinical Data Acquisition

**FIGURE 7:**
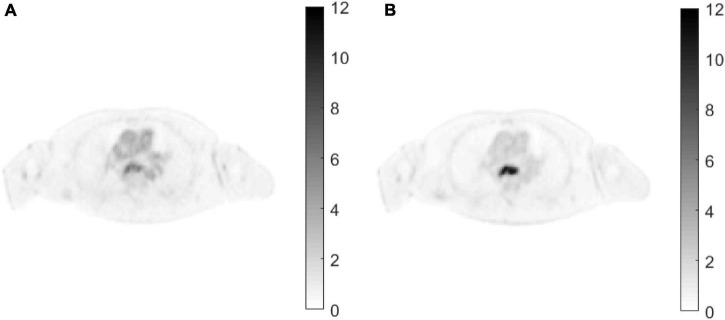
Clinical patient dynamic PET images. **(A)** Frame 22. **(B)** Frame 30.

Figure 7 shows two clinical patient Digital Imaging and Communications in Medicine (DICOM) dynamic PET images, which were collected from a uEXPLORER PET/CT imaging system (United Imaging Healthcare). Dynamic scanning was conducted for approximately 1 h after the injection of ^18^F-fluorodeoxyglucose (^18^F-FDG), and the dynamic PET data were divided into 30 frames: 6 × 5 s, 4 × 10 s, 4 × 30 s, 5 × 60 s, 4 × 180 s, and 8 × 300 s. The data were reconstructed as an image matrix of 192 × 192 × 673 voxels, with a slice sickness of 2.886 mm. The 3D list-mode ordered subset expectation maximization (OSEM) algorithm incorporated high-resolution time-of-flight (TOF) and point-spread function modeling (OSEM-TOF-PSF) with 3 iterations and 20 subsets (Zhang et al., 2017; Zhang X. et al., 2020). In this experiment, we used the data of patients with esophageal cancer. To observe the lesion more clearly, we selected 30 frames of the 200th slice. Forward projection was performed on the real PET image to obtain simulated projection data. Since the image reconstruction in the PET scanner involves random and scatter correction, we also added 20% of the projection data as the background (random and scattered events).

## Results

The dynamic PET sinogram data were rebinned into three composite frames with the same duration. The sinogram of the dynamic PET was obtained by forward projection of the DICOM images. All composite frames were reconstructed using the MLEM algorithm with 100 iterations.

**FIGURE 8:**
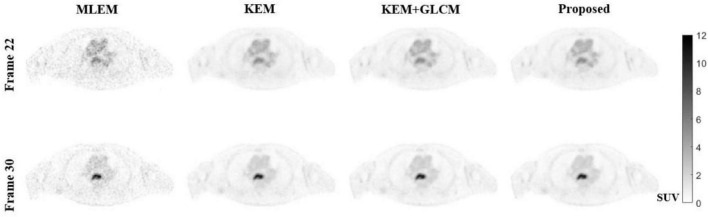
Reconstructed images of the 22nd and 30th frames by using different methods.

**FIGURE 9:**
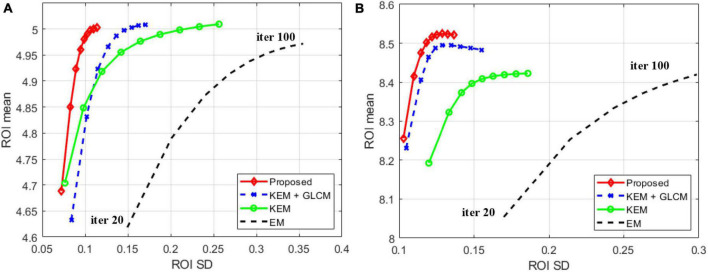
Mean activity versus SD trade-off of ROIs achieved by different methods for the **(A)** 22nd and **(B)** 30th frames.

Figure 8 shows the reconstructed image of the patient simulation data at the 15 million count level. Qualitatively, the overall image quality of the proposed method is higher than that of the KEM method and the expectation-maximization (EM) method. Compared with the texture of the image reconstructed by the KEM+GLCM method, the method proposed at the same time, the texture of the reconstructed image is closer to that of the real image. Figure 9 shows the quantitative calculations of the four reconstruction methods in high-uptake lesions. The results show that the image reconstructed by this method has a lower SD and a higher standardized uptake value (SUV) in the high-uptake lesion area; thus, this image is closer to the original image.

## Discussion

In this article, we use the KEM framework to incorporate the two different texture features of the prior image into the kernel function. The difference from the KEM+GLCM method is that we do not use the MRI image as the prior image but add multiple short time frames to obtain the composite frame reconstructed PET image as the prior image. There is no need to use other modal images. The difficulty of obtaining the prior images is reduced. Due to the use of two texture features, the reconstruction speed of the proposed algorithm is relatively slow.

To verify the feasibility of the proposed method, we conducted simulation experiments and conducted experiments using clinical data provided by the hospital. In Figure 3, we show reconstructed images from frames 12 and 24 by different reconstruction methods. This visualization allows an intuitive comparison of the images reconstructed by the proposed method with the images reconstructed by the compared methods. Compared to the other comparison algorithms, the proposed method produces better image quality. Figure 4 is a comparison of the NMSEs of different methods at each time frame. The NMSE is an index that reflects the difference between the reconstructed image and the real image. The figure shows that the proposed method has a lower NMSE in each time frame, thus indicating that the image reconstructed by the proposed method is the most similar to the real image. In Figure 5, we compare the CRC in the tumor area and the SD in the white matter area of several algorithms. The proposed method has the smallest SD in the white matter, and a higher CRC is obtained. The figure shows that the image reconstructed by the proposed algorithm has the smallest fluctuation in the intensity of the white matter, and the pixel value of the tumor can be close to the real image. In addition, we also verified this finding with Figure 6. The intensity of the image reconstructed by the proposed algorithm on the line passing through the tumor is closest to the real image. In addition, we also used clinical data for experiments. The simulation study of PET images of clinical patients also proves that the proposed method can achieve a better reconstruction effect in the tumor area.

The GLRLM and GGCM are used in the proposed algorithm. The GGCM adds edge information on the basis of the GLCM. The GLCM counts only the number of pixel pairs appearing in a certain direction, while the GGCM counts the pixel value and the gradient value common information. We also use the GLRLM, which reflects comprehensive image information on the direction, the adjacent interval, and the magnitude of change. Therefore, after using the features of the GLRLM and GGCM in the proposed algorithm, the reconstructed image outperforms the image reconstructed by the KEM+GLCM method in suppressing noise and retaining edges.

## Conclusion

In summary, we apply the new texture features to the KEM algorithm framework of PET reconstruction, and this method achieves better results in suppressing noise and improving the tumor contrast recovery coefficient.

## Data Availability Statement

The raw data supporting the conclusions of this article will be made available by the authors, without undue reservation.

## Ethics Statement

The studies involving human participants were reviewed and approved by Department of PET/CT, The First Affiliated Hospital of Shandong First Medical University, Shandong Provincial Qianfoshan Hospital, Jinan 250014, China. The patients/participants provided their written informed consent to participate in this study.

## Author Contributions

ZO and ZH conceived and designed the study. ZO, ZHC, and ZIC retrieved and analyzed the documents. ZO and ZHC wrote the manuscript. SZ, YD, NZ, DL, and ZH supervised the study and reviewed and edited the manuscript. All authors approved the final manuscript.

## Conflict of Interest

The authors declare that the research was conducted in the absence of any commercial or financial relationships that could be construed as a potential conflict of interest.

## Publisher’s Note

All claims expressed in this article are solely those of the authors and do not necessarily represent those of their affiliated organizations, or those of the publisher, the editors and the reviewers. Any product that may be evaluated in this article, or claim that may be made by its manufacturer, is not guaranteed or endorsed by the publisher.

## References

[B1] Cocosco C. A. Kollokian V. Kwan R. K.-S. Pike G. B. Evans A. C. (1997). Brainweb: online interface to a 3D MRI simulated brain database. *NeuroImage (Citeseer)* 5:425.

[B2] Dasarathy B. V. Holder E. B. (1991). Image characterizations based on joint gray level—run length distributions. *Pattern Recognit. Lett.* 12 497–502.

[B3] Delcroix O. Bourhis D. Keromnes N. Robin P. Le Roux P. Y. Abgral R. (2021). Assessment of image quality and lesion detectability with digital PET/CT system. *Front. Med.* 8:629096. 10.3389/fmed.2021.629096 33693016 PMC7937710

[B4] Doyen M. Mairal E. Bordonne M. Zaragori T. Roch V. Imbert L. (2021). Effect of point spread function deconvolution in reconstruction of brain 18F-FDG PET images on the diagnostic thinking efficacy in Alzheimer’s disease. *Front. Med.* 8:721551. 10.3389/fmed.2021.721551 34395486 PMC8358179

[B5] Galloway M. M. (1975). Texture analysis using gray level run lengths. *Comput. Graph. Image Process.* 4 172–179.

[B6] Gao D. Zhang X. Zhou C. Fan W. Zeng T. Yang Q. (2021). MRI-aided kernel PET image reconstruction method based on texture features. *Phys. Med. Biol.* 66:15NT03. 10.1088/1361-6560/ac1024 34192685

[B7] Green P. J. (1990). Bayesian reconstructions from emission tomography data using a modified EM algorithm. *IEEE Trans. Med. Imaging* 9 84–93. 10.1109/42.52985 18222753

[B8] Hu Z. Li Y. Zou S. Xue H. Sang Z. Liu X. (2020). Obtaining PET/CT images from non-attenuation corrected PET images in a single PET system using Wasserstein generative adversarial networks. *Phys. Med. Biol.* 65:215010. 10.1088/1361-6560/aba5e9 32663812

[B9] Jiang Y. Q. Gao Q. Chen H. Shi X. X. Wu J. B. Chen Y. (2021). Positron emission tomography-based short-term efficacy evaluation and prediction in patients with non-small cell lung cancer treated with hypo-fractionated radiotherapy. *Front. Oncol.* 11:590836. 10.3389/fonc.2021.590836 33718144 PMC7947869

[B10] Jiguang H. (1984). Gray level-gradient co-occurrence matrix texture analysis method. *Acta Automat. Sin.* 10 22–25.

[B11] Kang H. Kim E. E. Shokouhi S. Tokita K. Shin H. W. (2020). Texture analysis of f-18 fluciclovine PET/CT to predict biochemically recurrent prostate cancer: initial results. *Tomography* 6 301–307. 10.18383/j.tom.2020.00029 32879900 PMC7442090

[B12] Li L. Mu W. Wang Y. Liu Z. Liu Z. Wang Y. (2019). A non-invasive radiomic method using 18F-FDG PET predicts isocitrate dehydrogenase genotype and prognosis in patients with glioma. *Front. Oncol.* 9:1183. 10.3389/fonc.2019.01183 31803608 PMC6869373

[B13] Lubner M. G. Smith A. D. Sandrasegaran K. Sahani D. V. Pickhardt P. J. (2017). CT texture analysis: definitions, applications, biologic correlates, and challenges. *Radiographics* 37 1483–1503. 10.1148/rg.2017170056 28898189

[B14] Nuyts J. Beque D. Dupont P. Mortelmans L. (2002). A concave prior penalizing relative differences for maximum-a-posteriori reconstruction in emission tomography. *IEEE Trans. Nuclear Sci.* 49 56–60. 10.1109/TNS.2002.998681

[B15] Palumbo B. Bianconi F. Palumbo I. Fravolini M. L. Minestrini M. Nuvoli S. (2020). Value of shape and texture features from 18F-FDG PET/CT to discriminate between benign and malignant solitary pulmonary nodules: an experimental evaluation. *Diagnostics* 10:696. 10.3390/diagnostics10090696 32942729 PMC7555302

[B16] Ren J. Yuan Y. Qi M. Tao X. (2020). Machine learning–based CT texture analysis to predict HPV status in oropharyngeal squamous cell carcinoma: comparison of 2D and 3D segmentation. *Eur. Radiol.* 30 6858–6866. 10.1007/s00330-020-07011-4 32591885

[B17] Soydal C. Varli B. Araz M. Taskin S. Bakirarar B. Ortac F. (2021). Radiomics analysis of uterine tumors in 18F-flourodeoxyglucose positron emission tomography for prediction of lymph node metastases in endometrial carcinoma. *J. Nuclear Med.* 62:1519. 10.55730/1300-0144.5371 PMC10390124 36326312

[B18] Tang X. (1998). Texture information in run-length matrices. *IEEE Trans. Image Process.* 7 1602–1609. 10.1109/83.725367 18276225

[B19] Wady S. H. Yousif R. Z. Hasan H. R. A. (2020). Novel intelligent system for brain tumor diagnosis based on a composite neutrosophic-slantlet transform domain for statistical texture feature extraction. *BioMed Res. Int.* 2020:8125392. 10.1155/2020/8125392 32733955 PMC7369660

[B20] Wang G. (2019). High temporal-resolution dynamic PET image reconstruction using a new spatiotemporal kernel method. *IEEE Trans. Med. Imaging* 38 664–674. 10.1109/TMI.2018.2869868 30222553 PMC6422751

[B21] Wang G. Qi J. (2015). PET image reconstruction using kernel method. *IEEE Trans. Med. Imaging* 34 61–71. 10.1109/TMI.2014.2343916 25095249 PMC4280333

[B22] Wang Z. Bovik A. C. Sheikh H. R. Simoncelli E. P. (2004). Image quality assessment: from error visibility to structural similarity. *IEEE Trans. Image Process.* 13 600–612. 10.1109/tip.2003.819861 15376593

[B23] Zeng T. Gao J. Gao D. Kuang Z. Sang Z. Wang X. (2020). A GPU-accelerated fully 3D OSEM image reconstruction for a high-resolution small animal PET scanner using dual-ended readout detectors. *Phys. Med. Biol.* 65:245007. 10.1088/1361-6560/aba6f9 32679581

[B24] Zhang J. Zhao X. Zhao Y. Zhang J. Zhang Z. Wang J. (2020). Value of pre-therapy 18F-FDG PET/CT radiomics in predicting EGFR mutation status in patients with non-small cell lung cancer. *Eur. J. Nuclear Med. Mol. Imaging* 47 1137–1146. 10.1007/s00259-019-04592-1 31728587

[B25] Zhang W. Gao J. Yang Y. Liang D. Liu X. Zheng H. (2019). Image reconstruction for positron emission tomography based on patch-based regularization and dictionary learning. *Med. Phys.* 46 5014–5026. 10.1002/mp.13804 31494950 PMC6899708

[B26] Zhang X. Xie Z. Berg E. Judenhofer M. S. Liu W. Xu T. (2020). Total-body dynamic reconstruction and parametric imaging on the uexplorer. *J. Nuclear Med.* 61 285–291. 10.2967/jnumed.119.230565 31302637 PMC8801950

[B27] Zhang X. Zhou J. Cherry S. R. Badawi R. D. Qi J. (2017). Quantitative image reconstruction for total-body PET imaging using the 2-meter long EXPLORER scanner. *Phys. Med. Biol.* 62 2465–2485. 10.1088/1361-6560/aa5e46 28240215 PMC5524562

